# Multiobjective Optimization of a Frying Process Balancing Acrylamide Formation and Quality: Solution Analysis and Uncertainty Propagation

**DOI:** 10.3390/foods11223689

**Published:** 2022-11-17

**Authors:** Jose Lucas Peñalver-Soto, María Muñoz-Guillermo, Alberto Garre, Asunción Iguaz, Pablo S. Fernández, Jose A. Egea

**Affiliations:** 1 Departamento de Ingeniería de Alimentos y del Equipamiento Agrícola, Instituto de Biotecnología Vegetal, Universidad Politécnica de Cartagena (ETSIA), Paseo Alfonso XIII, 48, 30203 Cartagena, Spain; 2 Departamento de Mejora Vegetal, Centro de Edafología y Biología Aplicada del Segura (CEBAS-CSIC), Campus Universitario de Espinardo S/N, 30100 Murcia, Spain; 3 Departamento de Matemática Aplicada y Estadística, Universidad Politécnica de Cartagena, Antiguo Hospital de Marina (ETSII), Av. Dr. Fleming S/N, 30202 Cartagena, Spain

**Keywords:** multi-objective optimization, model-based optimization, equivalent solutions, uncertainty, Monte Carlo, frying operation, acrylamide, quality

## Abstract

In this study, we performed multi-objective model-based optimization of a potato-frying process balancing between acrylamide production and a quality parameter (yellowness). Solution analysis revealed that, for most of the Pareto solutions, acrylamide levels exceeded the EFSA recommendation. Almost equivalent optimal solutions were found for moderate processing conditions (low temperatures and/or processing times) and the propagation of the uncertainty of the acrylamide production model parameters led to Pareto fronts with notable differences from the one obtained using the nominal parameters, especially in the ranges of high values of acrylamide production and yellowness. These results can help to identify processing conditions to achieve the desired acrylamide/yellowness balance and design more robust processes allowing for the enhancement of flexibility when equivalent optimal solutions can be retrieved.

## 1. Introduction

### 1.1. Optimization in Food Engineering

Food engineering has become an increasingly important field, as evidenced by the growth of mathematical models devoted to understanding and improving food-processing operations [1]. One important application of mathematical modelling in food engineering is the optimization of food-processing operations.

Optimization is the process of finding the best possible solution to a problem. This usually involves finding the best compromise among several conflicting demands. To optimize a process, one must find the set of decision variables which, for example, maximize profitability while meeting a set of constraints. Several model-based optimization methods can be used to improve food processing. These methods are more rigorous than other empirical approaches and are thus more likely to find the best possible solution [2].

In general, optimization can be applied effectively to food processing if the changes during the process can be predicted mathematically. Heat, mass, and momentum transfers (as well as kinetics) are major mechanisms in food processing, and mathematical models describing these phenomena are essential for further mathematical-based optimization procedures [3].

Optimal operating conditions in the food industry are usually sought to ensure maximum profits and product quality, subject to constraints arising from food-safety issues and often environmental regulations. However, the dynamic, nonlinear and highly constrained nature of food-processing models can make the optimization of these processes a daunting task [4].

Achieving optimization in food processing requires some way of describing the potential alternatives and of choosing the best alternative. In the design, construction and maintenance of any engineering system, different technological and managerial decisions are required to be given at different stages of the process to either minimize the effort required or maximize the benefit desired. The formal description of any optimization problem has three parts [3]:

1. A set of variables that the optimization method can control and use to specify the alternatives (e.g., applying different process-temperature profiles during thermal processing to achieve better processing for a given objective function).
2. A set of requirements (e.g., the differential equations, boundary conditions, and integral equations specifying the constraints that the system and the variables are subjected to) that the optimization method must achieve or satisfy.
3. A measure of performance to compare one alternative to another (the objective function). The objective function, which may be continuous or in some cases discrete, is the function to be optimized (maximized or minimized). This can be accomplished by using either a mathematical model or by fitting an equation through the experimental data.

### 1.2. Applications of Multi-Objective Optimization in Food Engineering

For most industrial processes in food, simultaneous optimization of multiple objectives (e.g., product quality, operating costs, and safety) is the more realistic and desirable approach, but since these criteria are often opposing, the optimal solution is not unique. The multi-objective optimization (MOO) approach is used to find the best set of solutions for a problem with multiple objectives. In food engineering, MOO is used to optimize processes where conflicting objectives such as e.g., process economy, quality parameters or environmental indexes appear. These solutions are known as nondominated or Pareto optimal solutions [5]. Each of these solutions has no prior advantage over other Pareto optimal solutions so the objective of multi-objective optimization is to generate as many solutions as possible to evaluate and prioritize optimal trade-offs among the different objectives [6].

Multi-objective approaches have been used to solve optimization problems in the food engineering industry. For instance, Vilas et al. sought to maximize food quality and safety by developing smart active packaging systems that optimize food-packaging design and prediction of the expected shelf life along the food chain [7]. Abakarov used this technique with experimental data obtained on osmotic dehydration of carrot cubes in a sodium chloride solution to improve the assessment of criteria weights and produce fairer and more consistent products [6]. Holdsworth and Simpson obtained a set of Pareto-optimal solutions for processing time, quality retention, and texture loss under specific criteria of the processing temperature [8]. Krüger et al. proposed a multi-objective optimization to choose a pot and a growth substrate mixture such that environmental emissions and costs are simultaneously minimized [9] and Gergely et al. used this approach to improve wine filtration [10]. Sendín et al. used it to maximize the retention of several nutrients and quality factors and minimize the total process time [11]. Kiranoudis and Markatos considered the multi-objective approach to design the process of a conveyor-belt dryer using not only structural and operational process variables but the quality of treated potatoes [12]. In the same line, Olmos et al. used this approach to optimize the drying time maximizing the product quality [13] and Winiczenko et al. studied the effect of drying temperature and air velocity on apple quality parameters, such as color difference, volume ratio and water absorption capacity in convective drying [14]. In the field of sustainable distribution of foods, Bortolini et al. optimized the cost, delivery time and carbon footprint with a multi-objective approach [15]. However, no work was found where acrylamide production and food quality parameters were considered simultaneously in a multi-objective optimization approach. Peñalver-Soto et al. analysed the dynamics of acrylamide production and microbiological inactivation in certain foods by performing simulations instead of a formal optimization formulation [16].

### 1.3. Uncertainty in MOO

Uncertainty propagation has been extensively studied in the fields of physics and mathematics [17,18,19,20,21] and in particular, in the field of food intake, where it has been analyzed from different approaches [22,23,24,25,26,27]. In general, the propagation of uncertainty refers to the estimation of the variability in a given quantity. This variability can be due to several factors, such as measurement error, sampling variability, or natural variability [22]. In food engineering, the variability of a particular property or characteristic of a food product can have a significant impact on the quality and safety of the product.

In this study, the propagation of uncertainty to the MOO approach was assessed by propagating the uncertainty of model parameters to the solutions shown in the Pareto front. Parameter uncertainty can affect the shape of the Pareto optimal front in multi-objective optimization, and this can have important implications for decision-making [17].

Different methods can be used to propagate uncertainty in food engineering. Each method has its strengths and weaknesses, and the choice often depends on the type of data being studied. Some of the most common methods of uncertainty propagation include Monte-Carlo simulation [28], linear approximation [29], the sigma point method [30], and polynomial chaos expansion [31].

Specifically, this article applies the Monte-Carlo method which is a powerful tool for studying the propagation of uncertainty [32]. The method is used to calculate the probability of different outcomes by randomly selecting values from a probability distribution. This approach can be used to calculate the expected value of a function or to estimate the uncertainty in a measurement. The Monte-Carlo method can be used to study the propagation of uncertainty in food engineering. Garre et al. used this methodology in microbial inactivation of foods to select optimal experiment designs [33]. In this work, we analyzed the effects of parameter uncertainty in mathematical models describing food processes over the robustness of the Pareto set of solutions in multi-objective optimization using as a case study a frying process of potato chips where a quality parameter (yellowness) and the production of acrylamide, a potential carcinogen [34,35] were defined as opposed objectives.

## 2. Materials and Methods

### 2.1. Case Study

This study analyzed the potato-frying process to optimize food safety and culinary quality. Specifically, the impact of the heat treatment on the amount of acrylamide produced and the yellowness and moisture content were studied. The whole study was based on mathematical model simulations. We used the Maillard model (Equations (1)–(5)) to simulate the acrylamide formation using the fitted model provided by Knol et al. [36] and the models proposed by Krokida et al. [37,38] (Equations (6)–(11)) to simulate the yellowness and the moisture content. All models (see Section 2.2) were previously calibrated and validated by their original authors as reported in the respective bibliographic references The outputs of such models, which depend upon temperature and time, were used to simulate the experiments and perform the multi-objective optimization as well as the uncertainty propagation analysis. The simulated heat treatments considered were all in the range of values for which the mathematical models were validated according to their authors [36,37,38].

The selected potato corresponded to the Agria variety, which is used in products sold in supermarket chains and has been one of the most studied varieties in frying conditions in the literature [39,40,41]. The high temperatures of the considered heat treatment (frying process) brought the two considered objectives into conflict since an increase in yellowness implies an increase in acrylamide production, thus a multi-objective approach was used. Slices of 15 mm thickness were considered in this work. Likewise, no air drying or osmotic pre-treatment were considered [42]. The experiments carried out by Krokida et al. to calibrate the yellowness model used a commercial deep fat fryer with temperature control of ±1 °C that was filled with 2 l of oil and the potatoes-to-oil ratio was kept at 1:50 *w*/*v*. The concentration of hydrogenated cotton seed oil in total (refined plus hydrogenated) oil was considered as 50% [37]. Nevertheless, these authors stated that the type of oil did not have any influence over color parameters. They used a Hunterlab SAV colorimeter and reported the results in the CIE Lab color scale (non-dimensional) in their experiments [37,38]. Food safety was determined by low levels of acrylamide. EFSA [43] determines 50 μg/kg as the maximum level. On the other hand, culinary quality was determined by the maximization of yellowness and the setting of moisture content between 2 and 4% as recommended by Segnini et al. [44], as an indicator or predictor of texture.

**Figure 1:**
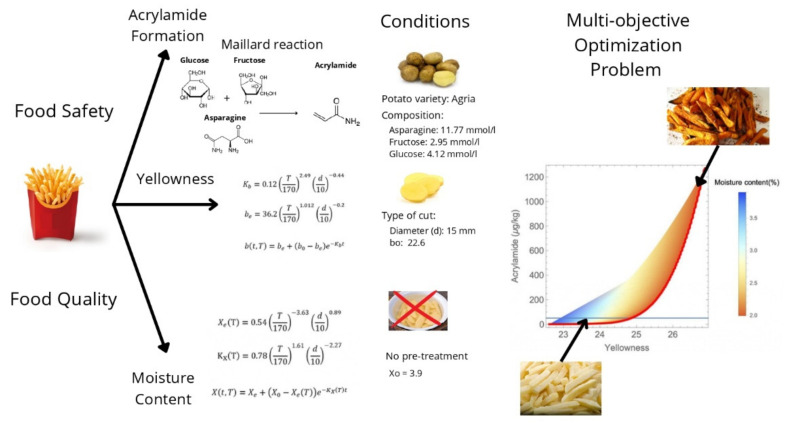
Outline of the case study.

If any of the described parameters took different values, a different potato variety was considered or additional quality variables (e.g., textural ones) were incorporated, and the methodology remained the same. Here we intend to illustrate how to design a frying process using modelling tools and, particularly, multi-objective optimization, as well as providing a global picture of the balances between objectives in the whole design variables domain, as shown in the visual scheme in Figure 1.

### 2.2. Mathematical Models

#### 2.2.1. Acrylamide Production

Because of its different applications in industry as a reactive molecule to synthesize polyacrylamide, acrylamide has been a focus of great interest [45,46]. Safe levels of exposure to acrylamide in human beings have been analyzed and studied. For a detailed review see [45] in which, among others, data on toxicology are included. EFSA in its latest report on the assessment of the genotoxicity of acrylamide [47], considered the possible modes of action of acrylamide carcinogenicity, including genotoxic and non-genotoxic effects. The paper concludes that there is substantial evidence for acrylamide genotoxicity mediated by metabolite formation, in addition to a possible contribution of non-genotoxic effects to acrylamide carcinogenicity. This is particularly interesting in food processes in which the heat treatment produces levels of acrylamide so large that they need to be controlled.

In this framework, different models have been considered. To quantify the acrylamide formation we used multi-response kinetics in a fructose–asparagine reaction at high temperatures (120–200 °C) proposed by Knol et al. [36]. The model is based on the reaction network shown in Equations (1) to (5).

Fructose and asparagine are degraded into glucose, acid acetic, Schiff base and unknown species (*X*_1_). At the same time, the Schiff base is degraded into melanoidins and acrylamide. Knol et al. fitted the equilibrium constants for the temperature range (120–200 °C) and showed a logarithmic relationship with temperature [36]. Therefore, the equilibrium constants for each temperature are calculated (*K_i_*(*T*) for each *i* = 1, 2,…, 6). The system of ordinary differential equations (ODEs) that relates the amount of acrylamide formed for a specific time *t* (minutes) and temperature *T*(°C) is defined in Equation (5).

(1) $$\frac{d\left[Glucose\right]}{dt}=-K_{1}\left(T\right)\cdot \left[Glucose\right]\cdot \left[Asparagine\right]-K_{2}\left(T\right)\cdot \left[Glucose\right]$$

(2) $$\frac{d\left[Fructose\right]}{dt}=-K_{3}\left(T\right)\cdot \left[Fructose\right]\cdot \left[Asparagine\right]+K_{2}\left(T\right)\cdot \left[Glucose\right]$$

(3) $$\frac{d\left[Asparagine\right]}{dt}=-K_{1}\left(T\right)\cdot \left[Glucose\right]\cdot \left[Asparagine\right]-K_{3}\left(T\right)\cdot \left[Fructose\right]\cdot \left[Asparagine\right]$$

(4) $$\frac{d\left[Schiff\;base\right]}{dt}=K_{1}\left(T\right)\cdot \left[Glucose\right]\cdot \left[Asparagine\right]+K_{3}\left(T\right)\cdot \left[Fructose\right]\cdot \left[Asparagine\right]-K_{4}\left(T\right)\cdot \left[Schiff\;base\right]\;-K_{5}\left(T\right)\cdot \left[Schiff\;base\right]$$

(5) $$\frac{d\left[Acrylamide\right]}{dt}=K_{4}\left(T\right)\cdot \left[Schiff\;base\right]-K_{6}\left(T\right)\cdot \left[Acrylamide\right]$$

One of the outputs of the ODEs is the acrylamide concentration formed for a heat treatment (time, temperature), which was the first optimization objective in our formulation. To solve this set of ODEs, apart from the heat treatment conditions, that is, the time and temperature variables, it is necessary to set the initial amounts of fructose, glucose, and asparagine. For the “Agria” potato, which was the variety studied, the compositions were 11.77 mmol/L of asparagine, 2.95 mmol/L of fructose and 4.12 mmol/L of glucose. Details on these calculations are provided in the Supplementary Materials [48,49].

#### 2.2.2. Yellowness

The second considered objective was a quality parameter related to the color of fried potato: yellowness. Pedreschi considered it as one of the quality parameters of interest for fried potatoes [50]. Color-related parameters are of great importance for the product to be attractive to the consumer [37,38,39,40,41,42,43,44,45,46,50,51]. In particular, different studies revealed that high values of yellowness are preferred by consumers [52]. The problem is that at the same time, an increase in temperature also implies an increase in another color-related parameter, redness, which is not a desirable quality in the final product [37]. Roughly speaking a good level of yellowness is “the goal” but, at the same time, redness must be minimized. Nevertheless, Knol et al. [36] and Pedreschi et al. [53] indicated that acrylamide concentration shows a good linear correlation with the redness of potato chips. Therefore redness can be indirectly controlled by the level of acrylamide (recall that the first objective considered in this work was to minimize the concentration of acrylamide). Here we used the model for the yellowness, namely *b*, proposed by Krokida et al. [37], described in Equation (6)–(8), where *d* is the thickness of the slice (mm) and T is the temperature (°C).

(6) $$K_{b}=0.12{\left(\frac{T}{170}\right)}^{2.49}{\left(\frac{d}{10}\right)}^{-0.44}$$

(7) $$b_{e}=36.2{\left(\frac{T}{170}\right)}^{1.012}{\left(\frac{d}{10}\right)}^{-0.2}$$

(8) $$b\left(t,\;T\right)=b_{e}+\left(b_{0}-b_{e}\right)e^{-K_{b}t}$$

Here we set the values of $d=15\;\mathrm{mm}\;\mathrm{and}\;\;b_{0}=22.6\;$ (corresponding to no pretreatment processes [37]). As observed in Equations (6)–(8), *b*, like our first-considered objective (acrylamide production), depends on both the time and temperature.

#### 2.2.3. Moisture Content

The moisture content of the fried product is an important quality parameter. Its control is necessary to achieve the desired taste, texture, and color of the product. The moisture content indicates the water loss from the potato strips during frying. It decreases significantly when the potato is fried. The temperature of the oil has a negative effect on the moisture content of fried potatoes. The higher the temperature of the frying oil, the lower the moisture content for the same frying time. Moisture content is also related to one of the quality aspects most valued by consumers, the degree of crispiness of fried potatoes. There is a direct relationship between these variables: the higher the moisture content, the lower the crispness. Therefore, it is of utmost importance that the moisture content value is maintained between 2% and 4% as recommended by Segnini et al. [44]. Following the model proposed by Krokida et al. [38], Equations (9)–(11) define the moisture content as a function of treatment time and temperature.

(9) $$X_{e}\left(T\right)=0.54{\left(\frac{T}{170}\right)}^{-3.63}{\left(\frac{d}{10}\right)}^{0.89}$$

(10) $$K_{X}\left(T\right)=0.78{\left(\frac{T}{170}\right)}^{1.61}{\left(\frac{d}{10}\right)}^{-2.27}$$

(11) $$X\left(t,T\right)=X_{e}+\left(X_{0}-X_{e}\left(T\right)\right)e^{-K_{X}\left(T\right)t}$$

where $X_{0}=3.9\;\mathrm{and}\;d=15\;\mathrm{mm},$ corresponding to no pretreatment, as indicated by Krokida et al. [38].

In our multi-objective optimization problem, we formulated the moisture content as a constraint whose value at the end of the frying process must lie between 2% and 4%, as recommended by Segnini et al. [44].

### 2.3. Multi-Objective Problem (MOP)

Multi-objective optimization aims at finding the best possible solutions to a set of conflicting objectives, Equations (12)–(16) define the mathematical formulation applied to our case study.

(12) $$\min_{u(t)}F\left(x\left(t\right),\;u\left(t\right)\right)$$

subject to:

(13) $$\frac{dx}{dt}=\Psi \left(x\left(t\right),\;u\left(t\right),\;t\right)$$

(14) $$x\left(t_{0}\right)=x_{0}$$

(15) g(x(t), u(t))≤0

(16) $$u^{L}\leq u\left(t\right)\leq u^{S}$$

where the vector of objective functions, Equation (12), contains all the objectives considered in the problem. In our case, the objectives were already defined as *f_1_* = acrylamide production (Equation (5)) and *f_2_* =−yellowness (Equation (8)), (note that the negative sign indicates that this objective is maximized). x is the vector of state variables (e.g., chemical species concentrations) and u is the vector of control variables (temperature and processing time in our case). Equation (13) represents the system dynamics (dynamic mathematical models that define acrylamide production and yellowness). Equation (14) represents the values of the stated variables at the beginning of the process (*t* = 0). Equation (15) represents inequality constraints, which can be considered at the end of the process or at intermediate times (moisture content in our MOP). Finally, Equation (16) corresponds to the lower and upper boundaries for the control variables (e.g., the minimum and maximum temperature and processing time). In our problem, those boundaries were defined as [0.1, 10] min for time and [120, 200] °C for temperature.

There are several methods for solving the problem. In the first approach, we used a systematic complete search using the nominal values for the model’s parameters to obtain not only the Pareto front but also the whole feasible region. The procedure is described as follows: A set *D* is defined as D:={(t,T) ∈[0.1, 10]×[120,200] :2≤X(t,T)≤4}, where *t* is the processing time, *T* is the temperature, and *X* is the moisture content. For every value b^*^ of yellowness within the interval [22.6, 26.9], which corresponds to the minimum and maximum yellowness values in the ranges of times and temperatures considered, we calculate the level curve $S_{b^{*}}:\;=\left\{\left(t,T\right)\;\in \left[0.1,\;10\right]\times \left[120,200\right]\;:\;b\left(t,T\right)=b^{*}\right\}\;\cap^{\;}D$. The numerical calculation of $S_{b^{*}}$ provides the feasible region and the values that minimize the acrylamide for every $S_{b_{i}}$ provide the Pareto front.

The described complete search procedure is computationally intensive since it evaluates all the solutions in the feasible regions. As stated above, this was only applied using the nominal parameter values. For assessing the uncertainty propagation, where 1000 Pareto fronts were calculated by simulating different values for the model parameters (see Section 2.4), the heuristic algorithm NSGA-II [54] was applied. Given the characteristics of the models considered in this study (nonlinear and dynamic), this type of algorithm is a suitable option to achieve good solutions (normally the optimal ones) in relatively short computational times [2]. Further, we checked that, for the nominal parameter values, the obtained Pareto front by NSGA-II coincided with that obtained with the complete search procedure. This study used the R package “nsga2” to perform the optimization of the proposed problem.

### 2.4. Uncertainty Propagation

Uncertainty propagation was applied to the estimation of the equilibrium constants of the differential equations describing acrylamide production (Equations (1)–(5)). The model considers up to six equilibrium constants for which confidence intervals are given in [36]. The Monte-Carlo method was used to simulate 1000 sets of positive parameter values following a normal distribution for each equilibrium constant The MOP was solved and a Pareto front for each combination of the simulated kinetic constants was obtained. Thus, 1000 Pareto fronts were obtained. These provide an idea of the uncertainty propagation of the equilibrium constants and their impact on the Pareto front.

## 3. Results

### 3.1. Multi-Objective Solutions

**Figure 2:**
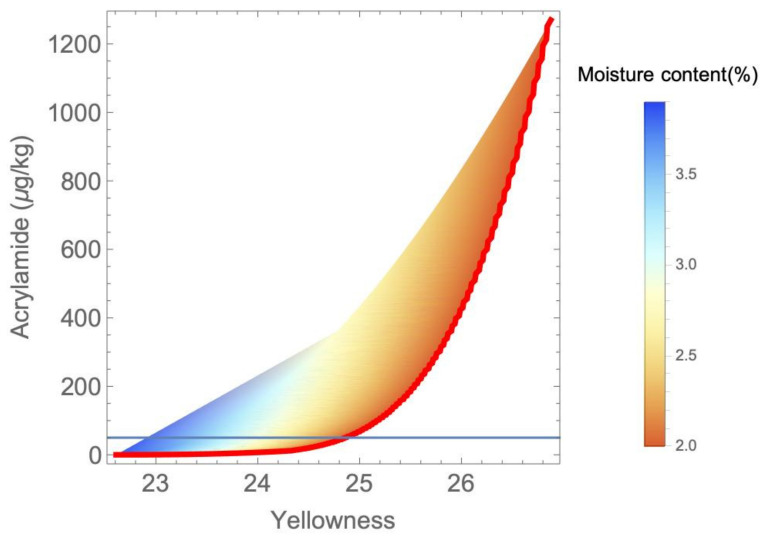
Feasible set of solutions and Pareto front.

The multi-objective approach using the nominal values for the kinetic parameters provided in [36] led to a set of optimal (non-dominated) solutions (Pareto front) shown in Figure 2 together with the feasible space. The vertical axis represents the amount of acrylamide produced and the horizontal axis represents the yellowness. The Pareto front is represented as a thick red line. On the other hand, the colors of the feasible region represent the moisture content and the blue horizontal line represents the recommended limit for acrylamide [43].

The first relationship was, as expected, that the higher the yellowness, the lower the moisture content and the higher the amount of acrylamide. All of this was positively correlated with the treatment severity (i.e., higher temperatures and/or treatment times led to an increase in the above-mentioned variables). On the other hand, given the problem boundaries and constraints, the yellowness was limited to values between 22 and 27, while the acrylamide did not exceed 1300 µg/kg which is 26 times higher than the EFSA recommendation.

**Figure 3:**
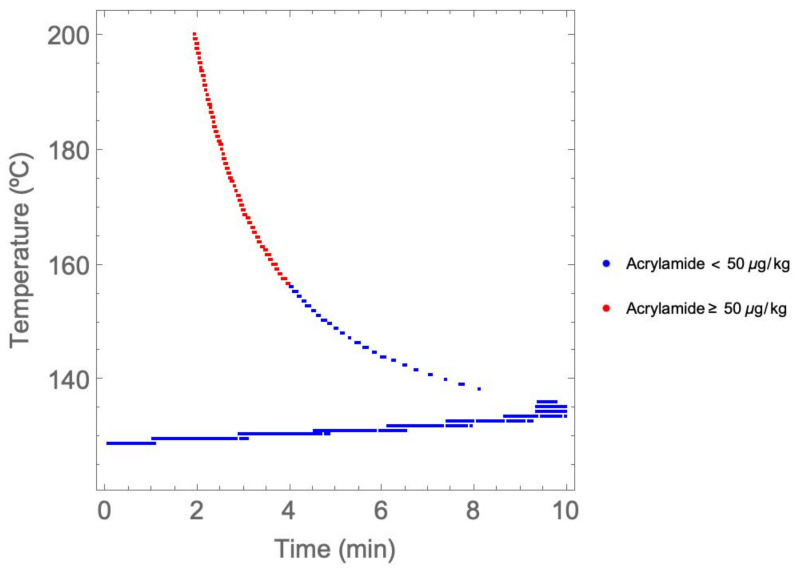
Pareto front temperature–time solutions.

A temperature–time representation of the Pareto front is shown in Figure 3. The points in red represent operational points that do not comply with the EFSA recommendation in terms of acrylamide amount, and they correspond to the highest temperatures. It can be seen that from approximately 155 °C, the dots form a curve that tends to be vertically asymptotic. This curve coincides with the conditions that keep the moisture content constraint active with a value of 2%.

As shown in Figure 2 and Figure 3, most of the solutions from the Pareto front led to high levels of acrylamide, exceeding the recommended levels by up to 26 times in some cases. Working points around 200 °C with a duration of approximately 2 min generated between 1200 and 1300 μg/kg of acrylamide when the recommended upper limit is 50 μg/kg. 

**Figure 4:**
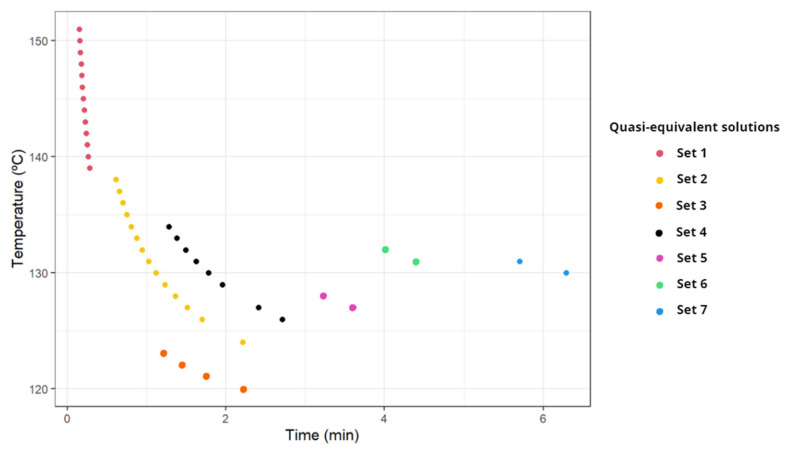
Selected sets of quasi-equivalent solutions.

During the optimization process, the existence of multiple quasi-equivalent solutions in the Pareto front was found for the ranges of low acrylamide production and low yellowness (e.g., low temperatures and/or processing times). The existence of these multiple solutions was caused by the flatness of the objective functions in areas of low temperatures and processing times (Figures S1 and S2). Table 1 illustrates some of these Pareto equivalent solutions. Equivalent solutions were defined as having the same values of acrylamide and yellowness with a tolerance of 0.01 but differences in temperature and time of at least 1 °C and 0.2 min, respectively. Figure 4 shows, in the temperature–time domain, the sets of equivalent solutions found for selected points of the Pareto front. Quasi-equivalent solutions are represented with the same color in Figure 4.

Table 1 and Figure 4 show that the lower the time and temperature, the higher number of equivalent solutions. As time or temperature increases, the number of equivalent solutions decreases and the curve defined by them becomes more horizontal (i.e., temperature differences are relatively lower than the difference in processing time in these cases). It is of note that, mathematically speaking, no equivalent solutions for the Pareto front can be found in this problem but, due to the flatness of the objective functions in certain temperature–time ranges, a set of very similar (called equivalent here) solutions can be found, allowing processes to be flexible to achieve certain results. No equivalent solutions according to the definition above were found for temperature-time conditions where acrylamide values were above 9 μg/kg.

### 3.2. Uncertainty Propagation

**Figure 5:**
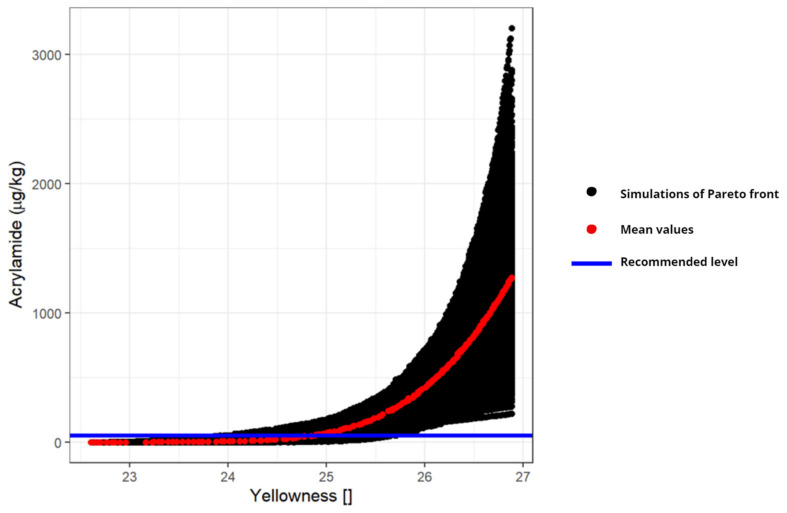
Set of 1000 Pareto fronts resulting from Monte-Carlo simulation of the kinetic parameters for the Maillard reaction.

In this section, we analyze the uncertainty propagation from the kinetic parameters of the Maillard equation characterized by Knol et al. [36] to the Pareto front of the multi-objective optimization problem. The result of the uncertainty propagation of the k’s of the Maillard model, Equations (1)–(5), with the Monte-Carlo method yields the set of Pareto fronts shown in Figure 5.

Figure 5 shows, on the vertical axis, the amount of acrylamide produced and on the horizontal axis, the yellowness. The black dots are the Pareto fronts of the 1000 simulations where the red line represents the Pareto front with the mean values of the Maillard’s kinetic parameters (shown in Figure 2) and the blue line represents the quantity of recommended acrylamide. The Pareto front resulting from the average kinetic values is located approximately in the middle zone of the solutions, so the assumed normal distributions for the kinetic parameters translate into a symmetric distribution of the Pareto solutions for each yellowness value. On the other hand, the uncertainty increases as the yellowness (i.e., temperature and/or time) increases. The combination of these two means that around 95% of the points considering all the 1000 Pareto fronts are outside the recommendation in terms of acrylamide production.

## 4. Discussion

This paper addresses the problem of food safety combined with product quality. It uses a multi-objective approach, which has been widely used in the literature [6,7,8,9,10,11,12,13,14,15]. Other studies such as that of Mestdagh [55] have studied balances between acrylamide and color but not from the quantitative and multi-objective optimization point of view addressed in this paper.

The MOP’s solutions (Figure 2) show that most frying processes (considering the conditions established in Section 2.2) do not comply with EFSA recommendations. The maximum acrylamide amount recommended by EFSA could be formulated as an additional constraint (which would lead to a different Pareto front) or, alternatively, we could try to select those points corresponding to temperatures not higher than 155 °C, approximately (blue points in Figure 3). Therefore, to ensure lower acrylamide values than the maximum ones recommended by EFSA, it is recommended to use frying temperatures below 160 °C with frying times not exceeding 4 min. The next implication is that, under these conditions, the yellowness only reaches values of 22–25, so the recommended amount of acrylamide greatly limits the visual quality of the final product regarding yellowness.

These results are influenced by all the premises taken such as the potato variety and the type of thermal process, among others. Therefore, any change in these assumptions may influence the results, although the procedure and analysis is useful for studying this type of problem. For example, Johnson determined that, given their composition, not only can the potato variety modify the balance between the variables, but the way they are grown can also have an influence [56].

On the other hand, these solutions are not unique since at the practical level equivalent solutions appear. Therefore, two frying processes with different conditions (time or temperature) can produce the same amount of acrylamide while maintaining equivalent quality (yellowness and moisture content). This existence of equivalent solutions in the Pareto front was recently observed by Ortiz-Martínez et al. [57] in the multi-objective optimization of a wastewater process. In any case, an a priori analysis of the objective functions and their dynamics can help to anticipate whether multiple solutions for the Pareto front can appear [16].

The study of the propagation of the uncertainty associated with the parameters complements a study that provides an additional tool to take into account other possible scenarios. The presented analysis shows that, when performing multi-objective optimization for design purposes, the model parameters variability and their propagation must be taken into account to find sets of design options (i.e., Pareto fronts) that account for every possible scenario. In this particular application, it was also shown that the propagated variability is not the same in every part of the objectives space, being lower with soft operating conditions (low values of yellowness and acrylamide production in Figure 2 than with severe operating conditions (high values of the objectives and higher process-temperature or time).

For a proper analysis of the optimal solutions, it must be taken into account that this modelling exercise considered that the cooking temperature is uniform throughout the potato and that the temperature of the oil is equal to the temperature of the potato. Obviously, this does not actually happen [58,59], so these theoretical times could be increased without affecting the limit of acrylamide produced. However, we would still conclude that most operating conditions within the ranges of temperatures and times usually considered in real processes exceed the recommended acrylamide amount. Finally, recall that this analysis used the mean values of the estimated kinetic parameters of the acrylamide production [36] but, considering the uncertainty of such parameters, other scenarios may occur. To account for this, we used uncertainty propagation tools to take into account other possible scenarios.

## 5. Conclusions

The multi-objective optimization of a potato-frying process balancing between acrylamide formation and a quality parameter (yellowness) was addressed in this study. The results show that most of the optimal solutions (the Pareto front) considering the usual temperature and processing time ranges provide higher acrylamide amounts than the limit recommended by EFSA (50 μg/kg). In addition, multiple solutions for some areas of the Pareto front (namely, those providing low values of acrylamide) have been identified. The existence of these multiple solutions can be anticipated by a previous analysis of the objective function and their sensitivities to changes in the decision variables (temperature and processing time) in different areas of the search space. In our case, both objective functions showed flat areas in the ranges of low temperatures and processing times, which allows the existence of multiple optimal solutions. These multiple solutions are not equivalent from a mathematical point of view but they are from a practical point of view, allowing us to slightly change the processing conditions to obtain the same results for the objective function within a given tolerance.

The uncertainty of the kinetic parameters for acrylamide production has been propagated to the Pareto front using a Monte-Carlo simulation, showing that the uncertainty with respect to the Pareto front using the nominal values increases as the values of the objective functions do. This uncertainty must be taken into account when designing the frying process to make the design more robust and avoid undesirable solutions (e.g., too high acrylamide values).

We recommend, if possible, performing these analyses when performing model-based multi-objective optimization to design food processes. This type of methodology is of course not specific to food processes but, given their nature, where multiple objectives must be optimized simultaneously, it should be applied to them. Other objectives could be included (e.g., other quality parameters or economic or environmental factors) or other types of food/processes where acrylamide production may be an issue can be considered by applying the methodologies presented here. These methodologies can help in making optimal decisions where there are unexpected conditions deviations or in re-designing the processes.

## Figures and Tables

**Table 1:** Selection of Pareto fronts and quasi-equivalent solutions.

Solutions	Time (min)	Temperature (°C)	Acrylamide (μg/kg)	Yellowness	Moisture Content (%)
#1	1.97	120.48	0.028	22.67	3.540
#1′	0.16	150.00	0.034	22.68	3.796
#2	1.99	124.74	0.069	22.76	3.425
#2′	0.61	138.00	0.076	22.76	3.614
#3	2.19	127.88	0.154	22.85	3.297
#3′	1.28	134.00	0.163	22.85	3.411
#4	2.05	132.91	0.345	22.96	3.199
#4′	3.61	127.00	0.348	22.96	3.060
#5	4.63	130.42	1.086	23.23	2.759
#5′	4.01	132.00	1.094	23.23	2.796
#6	5.03	132.39	1.821	23.39	2.617
#6′	6.29	130.00	1.823	23.39	2.571
#7	8.89	134.28	7.60	24.04	2.13
#7′	10.00	133.00	7.59	24.04	2.14

## Data Availability

The datasets generated for this study are available on request to the corresponding author.

## Supplementary Materials

The following supporting information can be downloaded at: https://www.mdpi.com/article/10.3390/foods11223689/s1. Figure S1: Surface plot of the acrylamide production (Objective function 1) with respect to processing time and temperature; Figure S2: Surface plot of the yellowness (Objective function 2) with respect to processing time and temperature.
