# Supplementary material for: Multiobjective Optimization of a Frying Process Balancing Acrylamide Formation and Quality: Solution Analysis and Uncertainty Propagation
Source: Foods. 2022 Nov 17;11(22):3689. doi: 10.3390/foods11223689 (PMC9689119; doi:10.3390/foods11223689)
Supplement: Supplementary file 1 [file foods-11-03689-s001.zip › foods-1959648-supplementary.pdf]

Multiobjective optimization of a frying process balancing acrylamide formation and quality: solution analysis and uncertainty propagation

Supplementary Material: Calculation of initial concentrations of glucose, fructose and asparagine for potato.

The *Agria* variety contains a significant quantity of glucose, asparagine and fructose of $3.89\,mmol/kg$, $11.10\,mmol/kg$ and $2.78\,mmol/kg$, respectively (Vivanti et al., 2006). If the density of the potato puree is considered to be $1.06\,kg/l$ (Golmohammadi & Afkari-Sayyah, 2012) then initial concentration of glucose is $C_{0,glucose} = 3.89\frac{mmol}{kg}\cdot 1.06\frac{kg}{l} = 4.12\,mM$, initial concentration of asparagine is $C_{0,asparagine} = 11.10\frac{mmol}{kg}\cdot 1.06\frac{kg}{l} = 11.77\,mM$ and initial concentration of fructose is $C_{0,fructose} = 2.78\frac{mmol}{kg}\cdot 1.06\frac{kg}{l} = 2.95\,mM$.

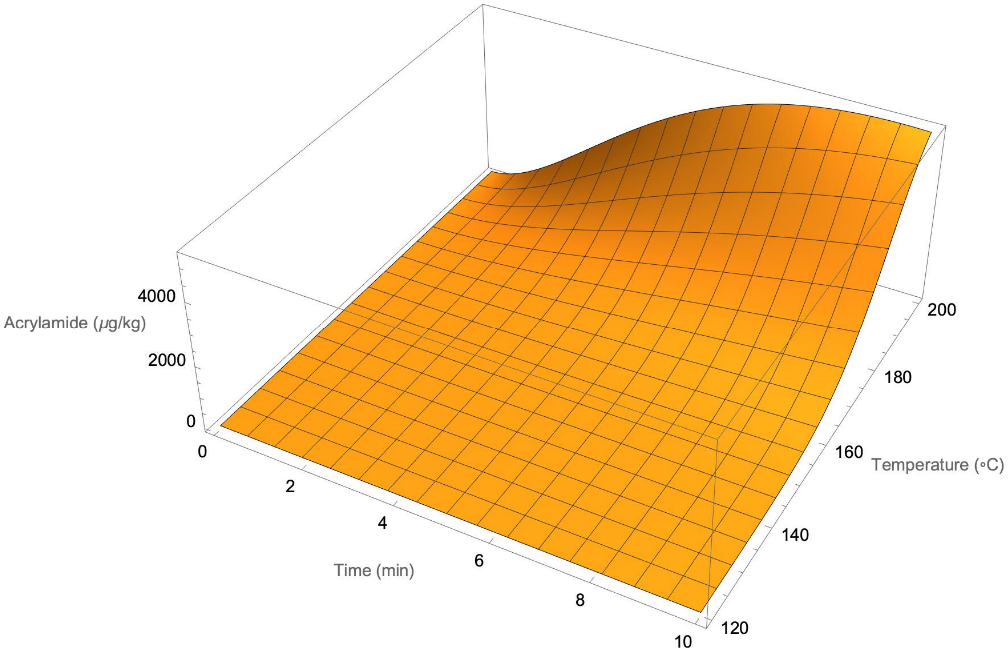

Figure S1: Surface plot of the acrylamide production (Objective function 1) with respect to processing time and temperature.

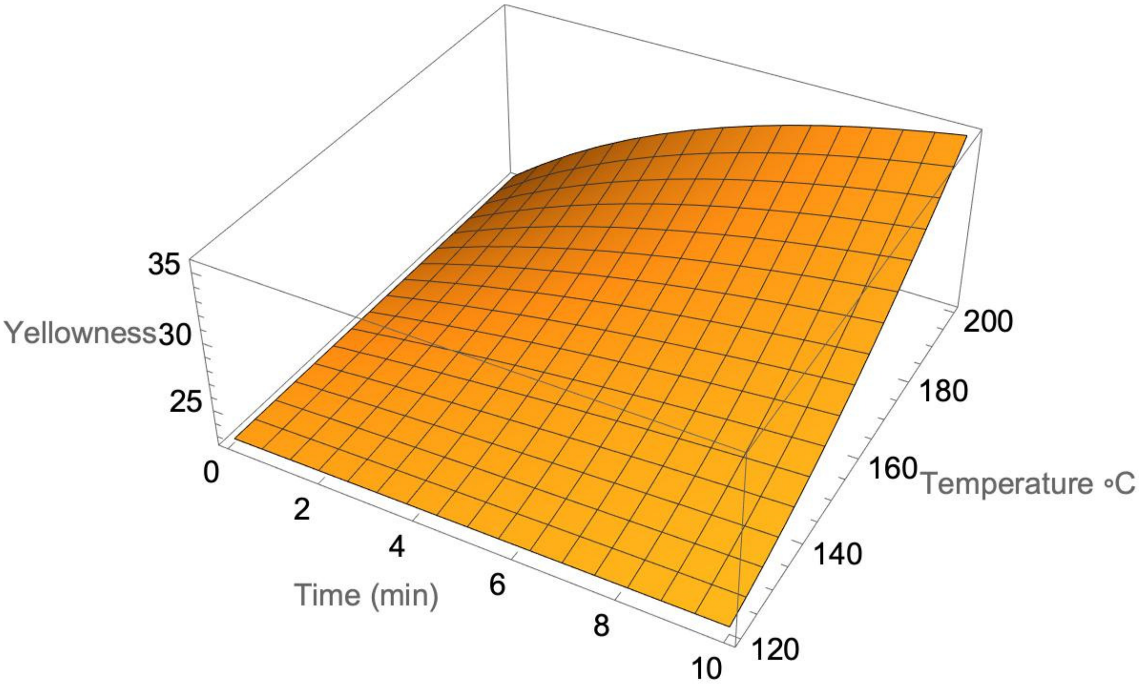

Figure S2: Surface plot of the yellowness (Objective function 2) with respect to processing time and temperature.

References

Golmohammadi, A., & Afkari-Sayyah, A. H. (2012). Long-Term Storage Effects on the Physical Properties of the Potato., 16(1), 104–113. https://doi.org/10.1080/10942912.2010.529978

Vivanti, V., Finotti, E., & Friedman, M. (2006). Level of Acrylamide Precursors Asparagine, Fructose, Glucose, and Sucrose in Potatoes Sold at Retail in Italy and in the United States. Journal of Food Science, 71(2), C81–C85. https://doi.org/10.1111/j.1365-2621.2006.tb08886.x
